# Why N,N-dimethyltryptamine matters: unique features and therapeutic potential beyond classical psychedelics

**DOI:** 10.3389/fpsyt.2024.1485337

**Published:** 2024-11-06

**Authors:** Cristiano Chaves, Rafael G. dos Santos, Serdar M. Dursun, Massimo Tusconi, Mauro Giovanni Carta, Elisa Brietzke, Jaime E. C. Hallak

**Affiliations:** ^1^ NeuroMood Lab, School of Medicine and Kingston Health Sciences Centre (KHSC), Department of Psychiatry, Queen’s University, Kingston, ON, Canada; ^2^ Department of Neuroscience and Behavior, Ribeirão Preto Medical School, University of São Paulo, São Paulo, Brazil; ^3^ National Institute for Translational Medicine (INCT-TM), CNPq, São Paulo, Brazil; ^4^ Department of Psychiatry (Neurochemical Research Unit) and Neuroscience and Mental Health Institute, University of Alberta, Edmonton, AB, Canada; ^5^ University Hospital of Cagliari, Cagliari, Italy; ^6^ Department of Medical Sciences and Public Health, University of Cagliari, Cagliari, Italy

**Keywords:** dimethyltryptamine (DMT), ayahuasca, psychedelics, hallucinogen, neuroplasticity, sigma-1 receptor, serotonin 2A (5HT2A) receptor, pharmacology

## Abstract

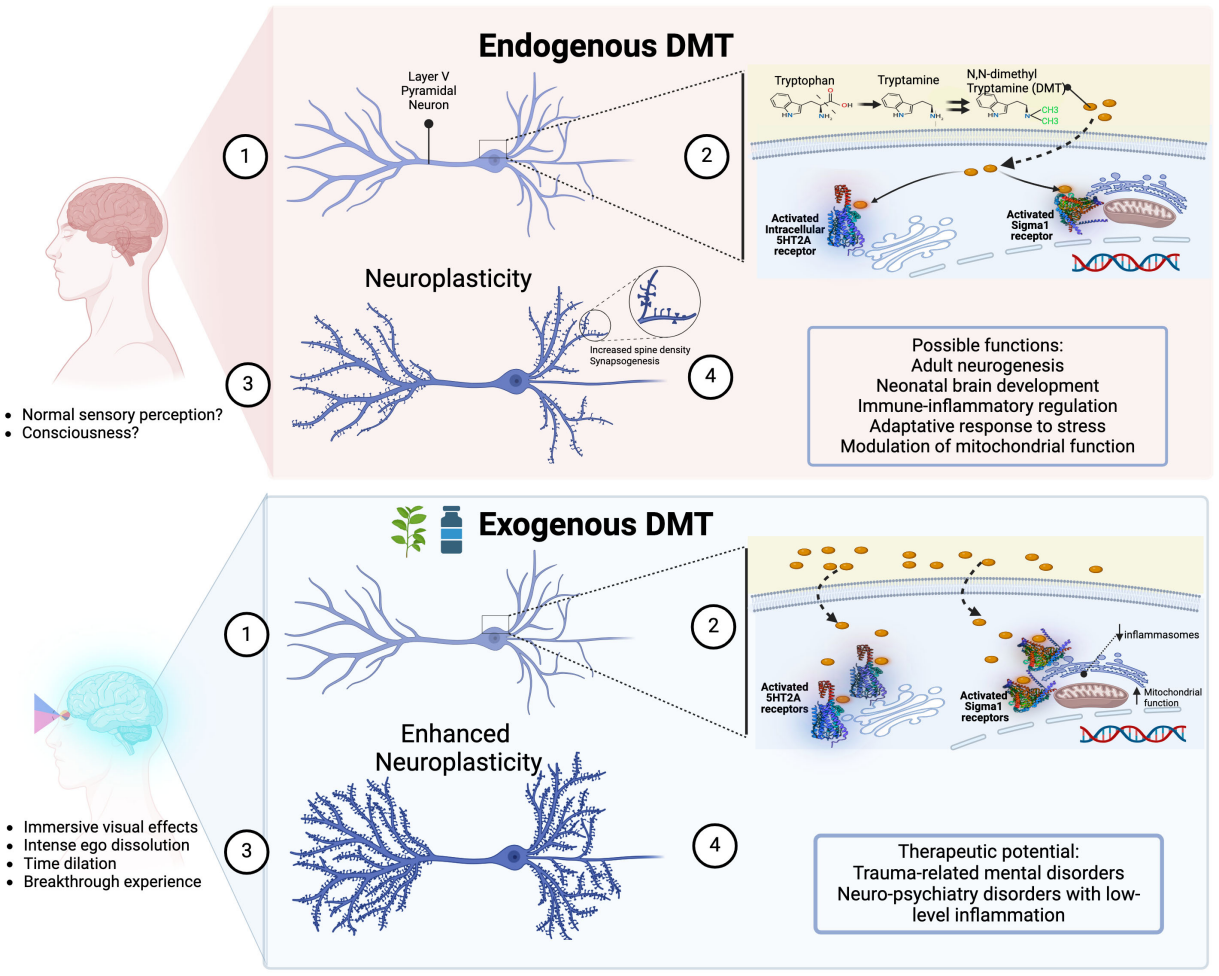

*‘Iracema comes with the pot full of the green liquor. The shaman decrees the dreams to each warrior and distributes the **wine of jurema**, which carries the brave Tabajara to heaven.’* ^1^

José de Alencar, in his poetic novel “Iracema” (1865)

## 1 Introduction

The “wine of jurema”, used in ancient Brazilian shamanic rituals, is rich in N,N-dimethyltryptamine (DMT), a naturally occurring tryptamine like serotonin and melatonin. Widely found in plants and animals, DMT is the main component of some botanical tisanes used for centuries as a channel of communication with the otherworld (1, 2). Despite being classified as a “classical psychedelic” (2–4), DMT’s unique effects are often overlooked due to an overemphasis on serotonin (5HT) 2A receptors as the key pharmacological feature of serotoninergic psychedelics. This simplification ignores DMT’s broader receptor interactions, lack of tolerance, and distinct subjective experiences. A nuanced understanding of DMT’s pharmacology and its redefinition among psychedelics is necessary to recognize its full potential.

DMT was originally synthesized by Canadian chemist Richard Manske in 1931, before it had ever been discovered in any plant (5), but its hallucinogenic properties were confirmed only in 1956 when chemist and psychiatrist Stephen Szara administered DMT intramuscularly to healthy volunteers, who experienced LSD-like effects (6). However, DMT had been identified earlier in *Mimosa hostilis* roots (the main component of the “wine of jurema”) by chemist O. Gonçalves de Lima in 1946 (7) and was later recognized in 1957 as a key psychoactive ingredient in ayahuasca (“vine of the souls”) (8).

These brews produce intense closed-eye (and, less frequently, open-eye) visual effects, known in Portuguese as “miração” (“seeings”), with immersive dream-like states and rich internal imagery (9–11). In contrast to other serotoninergic psychedelics, individuals who use ayahuasca and DMT report stronger visual effects, breakthrough experiences, near-death experiences, and encounters with entities (12–16).

This distinct phenomenology reflects DMT’s unique pharmacological profile, defying its simplistic classification as a classical psychedelic. In fact, DMT (a) activates sigma-1 receptors, trace amine-associated receptors (TAAR1), and intracellular 5HT2A receptors; (b) acts as a substrate of the serotonin uptake transporter (SERT) and the vesicle monoamine transporter (VMAT2); (c) and modulates dopaminergic, noradrenergic, adrenergic and cholinergic neurotransmission (1, 4, 17–19).

Unlike LSD, which induces complete tolerance after four consecutive daily doses (20), DMT’s effects remain minimally reduced with repeated use (21, 22). Moreover, DMT’s endogenous presence in the human body (urine, blood, cerebrospinal fluid) suggests roles in neuroplasticity, immune function, and other physiological processes, further distinguishing it from other psychedelics.

In mental health treatment, an exploratory study with intravenous DMT has shown next-day antidepressant effects in treatment-resistant depression (23). Ayahuasca has been long used in traditional Amazonian ceremonies with the aim of facilitating profound introspection and emotional healing. Modern preliminary research suggests its therapeutic potential for treating mood, anxiety, substance use, and trauma-related disorders (24–35), as well as suicidality (34) – possibly by modulating emotion and trauma processing (36–41).

However, these studies remain in their early stages, often involving small sample sizes and variability in methodologies. In contrast, LSD has a long tradition of studies, MDMA is approved in Australia for PTSD, and psilocybin, now also approved in Australia, shows promising potential for treatment-resistant depression. Hence, this study aims to highlight the unique characteristics of DMT that make it a promising candidate for psychedelic therapeutics.

## 2 DMT unique features

### 2.1 There is an endogenous production of DMT

A distinguishing feature of DMT is its natural production in the human body. Although often associated with hallucinogenic experiences when administered exogenously, DMT’s presence and role in the brain under normal physiological conditions remain an area of active investigation. Since the 1960s and 1970s we have known that mammals, including humans, endogenously produce DMT (18, 42, 43); for a comprehensive review, see Barker et al. (2012) (44). However, recent rodent studies show that DMT is present in the brain at levels akin to canonic neurotransmitters like serotonin and dopamine (45, 46).

Early research confirmed the presence of endogenous DMT in various tissues, including the liver and lungs, using techniques like gas chromatography and mass spectrometry (43). Traditionally, DMT synthesis have been attributed to the enzyme indolethylamine N-methyltransferase (INMT) (47). Nonetheless, Glynos et al. (2023) demonstrated that INMT is not essential for DMT production in rats, suggesting alternative enzymatic pathways (48).

Nichols (2018) critically examined the functional significance of endogenous DMT, particularly its secretion from the pineal gland and its link to near-death or out-of-body experiences (49). He argues that DMT concentrations in the brain are too low to produce psychoactive effects and emphasized the need for rigorous research.

Until 2018, few studies quantified DMT levels in rodent brains (50, 51), possibly losing sequestered DMT during tissue processing from whole-brain homogenates (17). However, Dean et al. (2019) provided substantial evidence of endogenous DMT in the rat brain (45), finding levels in the pineal gland and visual cortex comparable to other neuroamines. This suggests that DMT could be part of a functional system in normal brain physiology.

Dean et al. (2019) also observed a sudden increase in DMT levels in rats during cardiac arrest (45). However, Li et al. (2015) and Nichols (2018) (49, 52) noted that the time of death involves a “brainstorm” with a surge in neurotransmitters and synchronous electroencephalographic (EEG) signaling, indicative of high cognitive processing (53, 54), which aligns with experiences reported by cardiac arrest survivors.

Glynos et al. (2024) further explored DMT’s effects in animal models, finding that intravenous DMT administration in rats increased serotonin and dopamine levels, altered EEG spectral power, and enhanced functional connectivity (46). Importantly, they also detected endogenous DMT in the prefrontal and somatosensory cortices at levels comparable to serotonin and dopamine. These findings suggest that endogenous DMT may have functional significance in the mammalian brain (46), supporting previous results that DMT may accumulate and be stored in neuron vesicles (19, 55).

Although the natural role of DMT remains elusive, it has been suggested that DMT may be involved in diverse normal physiological functions: synaptic plasticity (56), neonatal brain development (50), adult neurogenesis regulation (57), normal sensory perception (58), modulation of brain mitochondrial function (59), adaptative immune response to stress (60–62), and protection against hypoxia and oxidative stress (63, 64).

### 2.2 DMT has an unique phenomenology

Compared to the other traditional psychedelics, DMT has a distinct phenomenology. When injected intravenously, DMT induces visions so strong that having one’s eyes open or closed barely affects what is seen (14). Breakthrough experiences, marked by profound changes in temporal and spatial perception, are common with ayahuasca and DMT, leading to feelings of being in a different reality, intense ego dissolution, and time dilation (15, 65, 66).

Near-death experiences (NDEs), featuring inner peace, out-of-body experiences, and exploration of otherworldly realms, closely resemble DMT-induced experiences. Accordingly, Timmermann et al. (2018) found striking parallels between actual NDEs and those induced by DMT (67).

A survey by Griffiths et al. (2019) on mystical experiences induced by classical psychedelics found that participants using DMT more often had complete mystical experiences, scoring higher on ineffability and transcendence of time and space compared to the use of psilocybin and LSD (68). In another survey by the same team, investigating interactions with sentient entities during DMT experience, 80% of respondents reported the experience profoundly altered their perception of reality, with 65% describing them as more real than typical waking consciousness (12).

Of note, although ayahuasca contains beta-carbolines, which act as monoamine oxidase inhibitors (MAOIs), its primarily psychedelic effect is mainly due to DMT. Beta-carbolines inhibit DMT’s metabolization by MAO enzymes in the gut and liver, allowing DMT to reach the brain and extending its effects from minutes to several hours (22, 69, 70). However, despite differences in duration and intensity, the experiences from ayahuasca are similar to those from exogenous DMT administration (71).

Interestingly, despite the molecular similarity between DMT and its derivative 5-methoxy-N,N-dimethyltryptamine (5-MeO-DMT), they have distinct characteristics. Both cause ego dissolution and time dilation, but 5-MeO-DMT often induces a sense of void or “whiteout”, contrasting with DMT’s vivid and intense visual phenomena (72).

### 2.3 DMT has unique pharmacokinetic properties

Tachyphylaxis, or rapid tolerance, is common with most classic serotoninergic psychedelics like LSD, psilocybin, and mescaline. Repeated administration of these substances quickly diminishes their subjective effects, usually within a few days, due to the downregulation and desensitization of extracellular cortical 5-HT2A receptors (73, 74). For instance, after four days of daily LSD administration, its hallucinogenic effects simply vanish (20, 75–79). Additionally, cross-tolerance between these substances is common; individuals tolerant to LSD, for example, show reduced sensitivity to psilocybin and mescaline, indicating shared mechanisms of action (80–88).

In contrast, DMT is unique in that it does not induce tolerance to its psychological effects, even with closely spaced repeated use. Studies in humans have consistently shown no significant attenuation in the subjective experiences elicited by DMT. For instance, Strassman (1995, 1996a) demonstrated that volunteers who received four closely spaced doses of DMT experienced no reduction in hallucinogenic intensity (21, 89). Similarly, Dos Santos et al. (2012) observed no tolerance to the subjective effects of two consecutive doses of ayahuasca (22). DMT also does not produce cross-tolerance to other hallucinogens like LSD, further highlighting its distinct pharmacodynamic properties (90–95). DMT’s unique lack of tolerance suggests a different mechanism of action compared to other serotoninergic psychedelics, making it a valuable compound for psychopharmacological research.

As with other psychedelics, there is the possibility of pharmacodynamic drug-drug interactions between DMT and other serotoninergic drugs, especially by competition at the receptor level (96). For instance, concomitant DMT administration with serotonin and norepinephrine reuptake inhibitors, or MAO inhibitors, may reduce DMT’s subjective effects by increasing serotonin levels and by downregulating 5-HT2A receptors after chronic use, and 5-HT2A antagonists may reduce the effects of DMT (96). Regarding possible toxicity in humans, human studies usually report a good tolerability profile (23), but elevations of cardiovascular parameters, anxiety, and unpleasant psychological reactions have been observed in clinical settings after acute DMT administration (4, 6, 14).

### 2.4 DMT has unique mechanisms of action

In 2009, Fontanilla and colleagues discovered that DMT is an endogenous ligand for sigma-1 receptors, found throughout the central nervous system and peripheral tissues (97). Initially mistaken for an opioid receptor, the sigma-1 receptor is now known as an orphan receptor because it binds synthetic compounds but not opioid peptides (97). Sigma-1 receptors act as transmembrane chaperone proteins, controlling anti-inflammatory reactions, cell survival, and neuronal differentiation (62). They have neurorestorative effects and protect cells against oxidative stress, underscoring their importance for brain health and function (60).

Unlike other classical psychedelics, DMT’s interaction with sigma-1 receptors may enhance neuroplasticity, neuroprotection and cognitive function. Cheng et al. (2024) showed that long-term DMT administration improved neurogenesis and cognitive function in rat models of Alzheimer’s disease by activating sigma-1 receptors, confirming its therapeutic potential in neurodegenerative disorders (59). Morales-Garcia et al. (2020) found that DMT promotes adult neurogenesis in the hippocampus via sigma-1 receptors, stimulating neural stem cell proliferation, neuroblast migration, and new neuron generation (57). These effects likely explain the improved spatial learning and memory observed in DMT-treated mice compared to controls (57).

DMT’s interaction with sigma-1 receptors also influences the immune system. DMT and its derivative 5-MeO-DMT modulate human monocyte-derived dendritic cells by activating sigma-1 receptors, reducing pro-inflammatory cytokine production and increasing anti-inflammatory cytokine secretion (62). This suggests that DMT may help maintain immune homeostasis and manage autoimmune and chronic inflammatory diseases. Additionally, DMT’s activation of sigma-1 receptors may offer therapeutic benefits in neuropsychiatric disorders characterized by low-level inflammation and cytokine imbalance (60).

### 2.5 DMT has unique effects in neuroplasticity

In a landmark study, Vargas et al. (2023) described how DMT and psilocybin activate intracellular cytoplasmic pools of 5HT2A receptors to promote neuroplasticity (98). This confirmed Cornea-Hébert et al. (1999) previous finding that 5HT2A receptors in the neuronal cortex are primarily intracellular rather than on the membrane surface (99). Remarkably, although serotonin is a potent 5HT2A receptor agonist, it cannot cross the cellular membrane to activate these receptors. However, the lipophilic nature of DMT allows it to cross cellular membranes and bind to these intracellular receptors, suggesting DMT, rather than serotonin, may be the endogenous agonist.

Moreover, downregulation and internalization of 5-HT2A receptors on the cell surface play a role in tolerance to psychedelics (20, 100–102). Interestingly, DMT’s action on intracellular pools of 5HT2A receptors may partially explain the lack of tolerance with repeated use (21). Accordingly, chronic DMT use does not induce 5HT2A receptor desensitization (2, 70).

Given its ability to promote neuroplasticity, DMT may be categorized as a psychoplastogen, a group of substances that may directly and rapidly change brain structure and function. This unique characteristic makes them promising therapeutic agents for neuropsychiatric disorders. By promoting dendritic growth and synapse formation, psychoplastogens like DMT may quickly alleviate symptoms of conditions such as anxiety and depression (103).

## 3 Discussion

DMT stands out among serotoninergic psychedelics for its potent visual effects, lack of tolerance, and unique neurophysiological properties. Its natural production and interaction with sigma-1 and intracellular 5HT2A receptors play important roles in brain plasticity and immune regulation. Evidence of substantial DMT levels in the rat brain, including its accumulation in neuron vesicles and alternative production pathways, suggests a broader role in neurobiology. While endogenous DMT is confirmed in the rat brain, its presence in the human brain and exact physiological roles need further exploration.

Despite its therapeutic potential, research on DMT faces key limitations, including uncertainty about the optimal dose and duration of therapeutic benefits, besides the challenge of functional unblinding due to its rapid and intense effects (23, 25). Variability in dosing, administrations routes, and the combination with a MAOI (in ayahuasca) further complicates cross-study comparisons (17). The long-term impacts of repeated dosing also require further investigation (104). It is crucial to have larger, well-designed trials to better understand DMT’s safety and efficacy (29, 30).

In addition to its unique pharmacological and therapeutic benefits, DMT could offer a cost-effective psychiatric treatment option if approved globally (39, 105, 106). Although the substance itself may not be patentable, like psilocybin, the processes and formulations used in different routes of administration (e.g. inhalation, intranasal, buccal or sublingual) could be, which could influence its accessibility (107). Nonetheless, DMT’s potential as a widely accessible treatment is significant, particularly given its potential to yield different treatment outcomes compared to other psychedelics, such as for neuropsychiatric disorders involving low-level inflammation.

DMT’s rapid onset and short duration (20-30 minutes when inhaled or injected) (17, 23) make it practical for clinical use compared to longer-acting psychedelics like psilocybin (4-6 hours), MDMA (4-6 hours), and LSD (8-12 hours) (108, 109). Its brief effects reduce supervision needs, and its lack of tolerance allows for repeated dosing. However, its short half-life and intense acute effects could complicate clinical use if frequent administration is needed, increasing demands on personnel and risk of adverse reactions (2, 3). Extended DMT infusion may address these limitations by offering more controlled, sustained effects (104, 110). While DMT shows promise in psychedelic therapy, more research is needed to explore its benefits, especially in combination with other molecules (111).

## Footnotes

1 Freely translated from the Portuguese; bold highlight not on the original.

## References

[1] Rossi GN Guerra LTL Baker GB Dursun SM Saiz JCB Hallak JEC. Molecular pathways of the therapeutic effects of ayahuasca, a botanical psychedelic and potential rapid-acting antidepressant. Biomolecules. (2022) 12:1618. doi: 10.3390/biom12111618 36358968 PMC9687782

[2] Cameron LP Olson DE. Dark classics in chemical neuroscience: N, N-dimethyltryptamine (DMT). ACS Chem Neurosci. (2018) 9:2344–57. doi: 10.1021/acschemneuro.8b00101 30036036

[3] Colosimo FA Borsellino P Krider RI Marquez RE Vida TA. The clinical potential of dimethyltryptamine: breakthroughs into the other side of mental illness, neurodegeneration, and consciousness. Psychoactives. (2024) 3:93–122. doi: 10.3390/psychoactives3010007

[4] Jimenez JH Bouso JC. Significance of mammalian N, N-dimethyltryptamine (DMT): A 60-year-old debate. J Psychopharmacol. (2022) 36:905–19. doi: 10.1177/02698811221104054 35695604

[5] Manske RH. A synthesis of the methyltryptamines and some derivatives. Can J Res. (1931) 5:592–600. doi: 10.1139/cjr31-097

[6] Szara S. Dimethyltryptamin: Its metabolism in man; the relation of its psychotic effect to the serotonin metabolism. Experientia. (1956) 12:441–2. doi: 10.1007/BF02157378 13384414

[7] Lima OG. Observações sobre o “vinho da Jurema” utilizado pelos índios Pancarú de Tacaratú (Pernambuco): Investigações complementares entre os Fulniô de Águas Belas (Pernambuco) e os remanescentes Tupís da Baía da Traição (Paraíba) [Potiguara]: Negerina: um alcaloide isolado da Mimosa hostilis Benth. Separata Arquivos Do Instituto Pesquisas Agronômicas (IPA). (1946) 4:45–80.

[8] Schultes RE. The identity of the malpighiaceous narcotics of South America. Botanical Museum Leaflets Harvard University. (1957) 18:1–56. doi: 10.5962/p.168508

[9] dos Santos RG Hallak JEC. Ayahuasca, an ancient substance with traditional and contemporary use in neuropsychiatry and neuroscience. Epilepsy Behav. (2021) 121:106300. doi: 10.1016/j.yebeh.2019.04.053 31182391

[10] dos Santos RG Enyart S Bouso JC Pares Ò Hallak JEC. Ayahuasca turned on my mind’s eye”: Enhanced visual imagery after ayahuasca intake in a man with “blind imagination” (aphantasia). J Psychedelic Stud. (2018) 2:74–7. doi: 10.1556/2054.2018.008

[11] de Araujo DB Ribeiro S Cecchi GA Carvalho FM Sanchez TA Pinto JP. Seeing with the eyes shut: neural basis of enhanced imagery following Ayahuasca ingestion. Hum Brain Mapp. (2012) 33:2550–60. doi: 10.1002/hbm.v33.11 PMC6870240 21922603

[12] Davis AK Clifton JM Weaver EG Hurwitz ES Johnson MW Griffiths RR. Survey of entity encounter experiences occasioned by inhaled N,N-dimethyltryptamine: Phenomenology, interpretation, and enduring effects. J Psychopharmacol. (2020) 34:1008–20. doi: 10.1177/0269881120916143 32345112

[13] Michael P Luke D Robinson O. An encounter with the self: A thematic and content analysis of the DMT experience from a naturalistic field study. Front Psychol. (2023) 14:1083356. doi: 10.3389/fpsyg.2023.1083356 37051610 PMC10083325

[14] Strassman RJ Qualls CR Uhlenhuth EH Kellner R. Dose-response study of N,N-dimethyltryptamine in humans. II. Subjective effects and preliminary results of a new rating scale. Arch Gen Psychiatry. (1994) 51:98–108. doi: 10.1001/archpsyc.1994.03950020022002 8297217

[15] Reckweg JT Uthaug MV Szabo A Davis AK Lancelotta R Mason NL. The clinical pharmacology and potential therapeutic applications of 5-methoxy-N,N-dimethyltryptamine (5-MeO-DMT). J Neurochem. (2022) 162:128–46. doi: 10.1111/jnc.v162.1 PMC9314805 35149998

[16] Rucker JJ Roberts C Seynaeve M Young AH Suttle B Yamamoto T. Phase 1, placebo-controlled, single ascending dose trial to evaluate the safety, pharmacokinetics and effect on altered states of consciousness of intranasal BPL-003 (5-methoxy-N,N-dimethyltryptamine benzoate) in healthy participants. J Psychopharmacol. (2024) 38(8):712–23. doi: 10.1177/02698811241246857 PMC11311898 38616411

[17] Barker SA. Administration of N,N-dimethyltryptamine (DMT) in psychedelic therapeutics and research and the study of endogenous DMT. Psychopharmacol (Berl). (2022) 239:1749–63. doi: 10.1007/s00213-022-06065-0 PMC8782705 35064294

[18] Barker SA Monti JA Christian STN. N-dimethyltryptamine: an endogenous hallucinogen. Int Rev Neurobiol. (1981) 22:83–110. doi: 10.1016/S0074-7742(08)60291-3 6792104

[19] Cozzi NV Gopalakrishnan A Anderson LL Feih JT Shulgin AT Daley PF. Dimethyltryptamine and other hallucinogenic tryptamines exhibit substrate behavior at the serotonin uptake transporter and the vesicle monoamine transporter. J Neural Transm (Vienna). (2009) 116:1591–9. doi: 10.1007/s00702-009-0308-8 19756361

[20] Nichols DE. Psychedelics. Pharmacol Rev. (2016) 68:264–355. doi: 10.1124/pr.115.011478 26841800 PMC4813425

[21] Strassman RJ Qualls CR Berg LM. Differential tolerance to biological and subjective effects of four closely spaced doses of N,N-dimethyltryptamine in humans. Biol Psychiatry. (1996) 39:784–95. doi: 10.1016/0006-3223(95)00200-6 8731519

[22] Dos Santos RG Grasa E Valle M Ballester MR Bouso JC Nomdedéu JF. Pharmacology of ayahuasca administered in two repeated doses. Psychopharmacol (Berl). (2012) 219:1039–53. doi: 10.1007/s00213-011-2434-x 21842159

[23] D’Souza DC Syed SA Flynn LT Safi-Aghdam H Cozzi NV Ranganathan M. Exploratory study of the dose-related safety, tolerability, and efficacy of dimethyltryptamine (DMT) in healthy volunteers and major depressive disorder. Neuropsychopharmacology. (2022) 47:1854–62. doi: 10.1038/s41386-022-01344-y PMC9372173 35660802

[24] Domínguez-Clavé E Soler J Pascual JC Elices M Franquesa A Valle M. Ayahuasca improves emotion dysregulation in a community sample and in individuals with borderline-like traits. Psychopharmacology. (2019) 236:573–80. doi: 10.1007/s00213-018-5085-3 30406413

[25] Dos Santos RG de Lima Osório F Rocha JM Rossi GN Bouso JC Rodrigues LS. Ayahuasca improves self-perception of speech performance in subjects with social anxiety disorder: A pilot, proof-of-concept, randomized, placebo-controlled trial. J Clin Psychopharmacol. (2021) 41:540–50. doi: 10.1097/JCP.0000000000001428 34166299

[26] Dos Santos RG Osório FL Crippa JA Hallak JE. Antidepressive and anxiolytic effects of ayahuasca: a systematic literature review of animal and human studies. Braz J Psychiatry. (2016) 38:65–72. doi: 10.1590/1516-4446-2015-1701 27111702 PMC7115465

[27] Giovannetti C Garcia Arce S Rush B Mendive F. Pilot evaluation of a residential drug addiction treatment combining traditional Amazonian medicine, ayahuasca and psychotherapy on depression and anxiety. J Psychoactive Drugs. (2020) 52:472–81. doi: 10.1080/02791072.2020.1789247 32748709

[28] Nunes AA Dos Santos RG Osório FL Sanches RF Crippa JA Hallak JE. Effects of ayahuasca and its alkaloids on drug dependence: A systematic literature review of quantitative studies in animals and humans. J Psychoactive Drugs. (2016) 48:195–205. doi: 10.1080/02791072.2016.1188225 27230395

[29] Osório Fde L Sanches RF Macedo LR Santos RG Maia-de-Oliveira JP Wichert-Ana L. Antidepressant effects of a single dose of ayahuasca in patients with recurrent depression: a preliminary report. Braz J Psychiatry. (2015) 37:13–20. doi: 10.1590/1516-4446-2014-1496 25806551

[30] Palhano-Fontes F Barreto D Onias H Andrade KC Novaes MM Pessoa JA. Rapid antidepressant effects of the psychedelic ayahuasca in treatment-resistant depression: a randomized placebo-controlled trial. psychol Med. (2019) 49:655–63. doi: 10.1017/S0033291718001356 PMC6378413 29903051

[31] Perkins D Pagni BA Sarris J Barbosa PC Chenhall R. Changes in mental health, wellbeing and personality following ayahuasca consumption: Results of a naturalistic longitudinal study. Front Pharmacol. (2022) 13:884703. doi: 10.3389/fphar.2022.884703 36386199 PMC9643165

[32] Sanches RF de Lima Osório F Dos Santos RG Macedo LR Maia-de-Oliveira JP Wichert-Ana L. Antidepressant effects of a single dose of ayahuasca in patients with recurrent depression: a SPECT study. J Clin Psychopharmacol. (2016) 36:77–81. doi: 10.1097/JCP.0000000000000436 26650973

[33] Santos RG Landeira-Fernandez J Strassman RJ Motta V Cruz AP. Effects of ayahuasca on psychometric measures of anxiety, panic-like and hopelessness in Santo Daime members. J Ethnopharmacol. (2007) 112:507–13. doi: 10.1016/j.jep.2007.04.012 17532158

[34] Zeifman RJ Singhal N Dos Santos RG Sanches RF de Lima Osório F Hallak JE. Rapid and sustained decreases in suicidality following a single dose of ayahuasca among individuals with recurrent major depressive disorder: results from an open-label trial. Psychopharmacology. (2021) 238:453–9. doi: 10.1007/s00213-020-05692-9 33118052

[35] Van Oorsouw K Toennes SW Ramaekers JG. Therapeutic effect of an ayahuasca analogue in clinically depressed patients: a longitudinal observational study. Psychopharmacology. (2022) 239:1839–52. doi: 10.1007/s00213-021-06046-9 PMC8785027 35072760

[36] Argento E Capler R Thomas G Lucas P Tupper KW. Exploring ayahuasca-assisted therapy for addiction: A qualitative analysis of preliminary findings among an Indigenous community in Canada. Drug Alcohol Review. (2019) 38:781–9. doi: 10.1111/dar.12985 31489731

[37] Hamill J Hallak J Dursun SM Baker G. Ayahuasca: psychological and physiologic effects, pharmacology and potential uses in addiction and mental illness. Curr Neuropharmacol. (2019) 17:108–28. doi: 10.2174/1570159X16666180125095902 PMC6343205 29366418

[38] Loizaga-Velder A. A psychotherapeutic view on therapeutic effects of ritual ayahuasca use in the treatment of addiction. MAPS Bull. (2013) 23:36–40.

[39] Palhano-Fontes F Soares BL Galvão-Coelho NL Arcoverde E Araujo DB. Ayahuasca for the treatment of depression. Disruptive Psychopharmacol: Springer. (2021) p:113–24. doi: 10.1007/7854_2021_277 34761362

[40] Perkins D Ruffell SG Day K Pinzon Rubiano D Sarris J. Psychotherapeutic and neurobiological processes associated with ayahuasca: A proposed model and implications for therapeutic use. Front Neurosci. (2023) 16:879221. doi: 10.3389/fnins.2022.879221 36798604 PMC9928213

[41] Raut SB Marathe PA van Eijk L Eri R Ravindran M Benedek DM. Diverse therapeutic developments for post-traumatic stress disorder (PTSD) indicate common mechanisms of memory modulation. Pharmacol Ther. (2022) 239:108195. doi: 10.1016/j.pharmthera.2022.108195 35489438

[42] Christian ST Harrison R Quayle E Pagel J Monti J. The *in vitro* identification of dimethyltryptamine (DMT) in mammalian brain and its characterization as a possible endogenous neuroregulatory agent. Biochem Med. (1977) 18:164–83. doi: 10.1016/0006-2944(77)90088-6 20877

[43] Franzen F Gross H. Tryptamine N N-dimethyltryptamine N. N-dimethyl-5-hydroxytryptamine and 5-methoxytryptamine in human blood and urine. Nature. (1965) 206:1052–. doi: 10.1038/2061052a0 5839067

[44] Barker SA McIlhenny EH Strassman R. A critical review of reports of endogenous psychedelic N, N-dimethyltryptamines in humans: 1955-2010. Drug Test Anal. (2012) 4:617–35. doi: 10.1002/dta.v4.7-8 22371425

[45] Dean JG Liu T Huff S Sheler B Barker SA Strassman RJ. Biosynthesis and extracellular concentrations of N,N-dimethyltryptamine (DMT) in mammalian brain. Sci Rep. (2019) 9:9333. doi: 10.1038/s41598-019-45812-w 31249368 PMC6597727

[46] Glynos NG Huels ER Nelson A Kim Y Kennedy RT Mashour GA. Neurochemical and neurophysiological effects of intravenous administration of N,N -dimethyltryptamine in rats. bioRxiv. (2024). doi: 10.1101/2024.04.19.589047 PMC12873648 41419333

[47] Axelrod J. Enzymatic formation of psychotomimetic metabolites from normally occurring compounds. Science. (1961) 134:343–. doi: 10.1126/science.134.3475.343 13685339

[48] Glynos NG Carter L Lee SJ Kim Y Kennedy RT Mashour GA. Indolethylamine N-methyltransferase (INMT) is not essential for endogenous tryptamine-dependent methylation activity in rats. Sci Rep. (2023) 13:280. doi: 10.1038/s41598-023-27538-y 36609666 PMC9822953

[49] Nichols DE. N,N-dimethyltryptamine and the pineal gland: Separating fact from myth. J Psychopharmacol. (2018) 32:30–6. doi: 10.1177/0269881117736919 29095071

[50] Beaton JM Morris PE. Ontogeny of N, N-dimethyltryptamine and related indolealkylamine levels in neonatal rats. Mech Ageing Dev. (1984) 25:343–7. doi: 10.1016/0047-6374(84)90007-1 6588281

[51] Kärkkäinen J Forsström T Tornaeus J Wähälä K Kiuru P Honkanen A. Potentially hallucinogenic 5-hydroxytryptamine receptor ligands bufotenine and dimethyltryptamine in blood and tissues. Scandinavian J Clin Lab Invest. (2005) 65:189–99. doi: 10.1080/00365510510013604 16095048

[52] Li D Mabrouk OS Liu T Tian F Xu G Rengifo S. Asphyxia-activated corticocardiac signaling accelerates onset of cardiac arrest. Proc Natl Acad Sci. (2015) 112:E2073–E82. doi: 10.1111/nyas.2015.1362.issue-1 PMC4413312 25848007

[53] Borjigin J Lee U Liu T Pal D Huff S Klarr D. Surge of neurophysiological coherence and connectivity in the dying brain. Proc Natl Acad Sci. (2013) 110:14432–7. doi: 10.1073/pnas.1308285110 PMC3761619 23940340

[54] Nichols CD Nichols DE. DMT in the mammalian brain: A critical appraisal. ALIUS Bulletin. (2020) 4:16–22. doi: 10.34700/s66k-9j57

[55] Sangiah S Gomez M Domino E. Accumulation of N, N-dimethyltryptamine in rat brain cortical slices. Biol Psychiatry. (1979) 14:925–36. 41604

[56] Carbonaro TM Gatch MB. Neuropharmacology of N,N-dimethyltryptamine. Brain Res Bull. (2016) 126:74–88. doi: 10.1016/j.brainresbull.2016.04.016 27126737 PMC5048497

[57] Morales-Garcia JA Calleja-Conde J Lopez-Moreno JA Alonso-Gil S Sanz-SanCristobal M Riba J. N,N-dimethyltryptamine compound found in the hallucinogenic tea ayahuasca, regulates adult neurogenesis *in vitro* and *in vivo* . Transl Psychiatry. (2020) 10:331. doi: 10.1038/s41398-020-01011-0 32989216 PMC7522265

[58] Wallach J. Endogenous hallucinogens as ligands of the trace amine receptors: a possible role in sensory perception. Med Hypotheses. (2009) 72:91–4. doi: 10.1016/j.mehy.2008.07.052 18805646

[59] Cheng D Lei Z-G Chu K Lam OJH Chiang CY Zhang Z-JN. N-Dimethyltryptamine, a natural hallucinogen, ameliorates Alzheimer’s disease by restoring neuronal Sigma-1 receptor-mediated endoplasmic reticulum-mitochondria crosstalk. Alzheimer’s Res Ther. (2024) 16:95. doi: 10.1186/s13195-024-01462-3 38693554 PMC11061967

[60] Frecska E Bokor P Winkelman M. The therapeutic potentials of ayahuasca: possible effects against various diseases of civilization. Front Pharmacol. (2016) 7:35. doi: 10.3389/fphar.2016.00035 26973523 PMC4773875

[61] Frecska E Szabo A Winkelman MJ Luna LE McKenna DJ. A possibly sigma-1 receptor mediated role of dimethyltryptamine in tissue protection, regeneration, and immunity. J Neural Transm (Vienna). (2013) 120:1295–303. doi: 10.1007/s00702-013-1024-y 23619992

[62] Szabo A Kovacs A Frecska E Rajnavolgyi E. Psychedelic N,N-dimethyltryptamine and 5-methoxy-N,N-dimethyltryptamine modulate innate and adaptive inflammatory responses through the sigma-1 receptor of human monocyte-derived dendritic cells. PloS One. (2014) 9:e106533. doi: 10.1371/journal.pone.0106533 25171370 PMC4149582

[63] Szabo A Kovacs A Riba J Djurovic S Rajnavolgyi E Frecska E. The Endogenous Hallucinogen and Trace Amine N,N-Dimethyltryptamine (DMT) Displays Potent Protective Effects against Hypoxia via Sigma-1 Receptor Activation in Human Primary iPSC-Derived Cortical Neurons and Microglia-Like Immune Cells. Front Neurosci. (2016) 10:423. doi: 10.3389/fnins.2016.00423 27683542 PMC5021697

[64] Szabo I Varga VE Dvoracsko S Farkas AE Kormoczi T Berkecz R. N,N-Dimethyltryptamine attenuates spreading depolarization and restrains neurodegeneration by sigma-1 receptor activation in the ischemic rat brain. Neuropharmacology. (2021) 192:108612. doi: 10.1016/j.neuropharm.2021.108612 34023338

[65] Fradkin D. Breaking through the doors of perception, consciousness, and existence: to what extent does psychedelic phenomenology ontologically depend on external factors? J Psychedelic Stud. (2024) 8:122–41. doi: 10.1556/2054.2022.00168

[66] Gallimore A. Building alien worlds—the neuropsychological and evolutionary implications of the astonishing psychoactive effects of N,N-dimethyltryptamine (DMT). J Sci Exploration. (2013) 27:455–503.

[67] Timmermann C Roseman L Williams L Erritzoe D Martial C Cassol H. DMT models the near-death experience. Front Psychol. (2018) 9:395026. doi: 10.3389/fpsyg.2018.01424 PMC6107838 30174629

[68] Griffiths RR Hurwitz ES Davis AK Johnson MW Jesse R. Survey of subjective “God encounter experiences”: Comparisons among naturally occurring experiences and those occasioned by the classic psychedelics psilocybin, LSD, ayahuasca, or DMT. PloS One. (2019) 14:e0214377. doi: 10.1371/journal.pone.0214377 31013281 PMC6478303

[69] Riba J Valle M Urbano G Yritia M Morte A Barbanoj MJ. Human pharmacology of ayahuasca: subjective and cardiovascular effects, monoamine metabolite excretion, and pharmacokinetics. J Pharmacol Exp Ther. (2003) 306:73–83. doi: 10.1124/jpet.103.049882 12660312

[70] Smith RL Canton H Barrett RJ Sanders-Bush E. Agonist properties of N, N-dimethyltryptamine at serotonin 5-HT2A and 5-HT2C receptors. Pharmacol Biochem Behav. (1998) 61:323–30. doi: 10.1016/S0091-3057(98)00110-5 9768567

[71] Ramaekers JG Mallaroni P Kloft L Reckweg JT Toennes SW van Oorsouw K. Altered state of consciousness and mental imagery as a function of N, N-dimethyltryptamine concentration in ritualistic ayahuasca users. J Cognit Neurosci. (2023) 35:1382–93. doi: 10.1162/jocn_a_02003 37159257

[72] Dourron HM Nichols CD Simonsson O Bradley M Carhart-Harris R Hendricks PS. 5-MeO-DMT: An atypical psychedelic with unique pharmacology, phenomenology & risk? Psychopharmacol (Berl). (2023). doi: 10.1007/s00213-023-06517-1 38072874

[73] de la Fuente Revenga M Jaster AM McGinn J Silva G Saha S González-Maeso J. Tolerance and cross-tolerance among psychedelic and nonpsychedelic 5-HT(2A) receptor agonists in mice. ACS Chem Neurosci. (2022) 13:2436–48. doi: 10.1021/acschemneuro.2c00170 PMC10411500 35900876

[74] Wallach J Cao AB Calkins MM Heim AJ Lanham JK Bonniwell EM. Identification of 5-HT(2A) receptor signaling pathways associated with psychedelic potential. Nat Commun. (2023) 14:8221. doi: 10.1038/s41467-023-44016-1 38102107 PMC10724237

[75] Abramson HA Jarvik ME Gorin M Hirsch M. Lysergic acid diethylamide (LSD-25): XVII. Tolerance development and its relationship to a theory of psychosis. J Psychol. (1956) 41:81–105. doi: 10.1080/00223980.1956.9916206

[76] Belleville RE Fraser HF Isbell H Logan CR Wikler A. Studies on lysergic acid diethylamide (LSD-25). I. Effects in former morphine addicts and development of tolerance during chronic intoxication. AMA Arch Neurol Psychiatry. (1956) 76:468–78. doi: 10.1001/archneurpsyc.1956.02330290012002 13371962

[77] Cholden LS Kurland A Savage C. Clinical reactions and tolerance to LSD in chronic schizophrenia. J Nervous Ment Dis. (1955) 122:211–21. doi: 10.1097/00005053-195509000-00001 13295823

[78] Commissaris R Lyness W Cordon J Moore K Rech R. Behavioral tolerance to the effects of LSD in the rat. Subst Alcohol Actions/misuse. (1980) 1:203–7. 7302781

[79] Freedman DX Aghajanian GK Ornitz EM Rosner BS. Patterns of tolerance to lysergic acid diethylamide and mescaline in rats. Science. (1958) 127:1173–4. doi: 10.1126/science.127.3307.1173 13555858

[80] Abramson HA Rolo A Sklarofsky B Stache J. Production of cross-tolerance to psychosis-producing doses of lysergic acid diethylamide and psilocybin. J Psychol. (1960) 49:151–4. doi: 10.1080/00223980.1960.9916396

[81] Appel J Freedman D. Tolerance and cross-tolerance among psychotomimetic drugs. Psychopharmacologia. (1968) 13:267–74. doi: 10.1007/BF00401404 5679628

[82] Balestrieri A Fontanari D. Acquired and crossed tolerance to mescaline, LSD-25, and BOL-148. AMA Arch Gen Psychiatry. (1959) 1:279–82. doi: 10.1001/archpsyc.1959.03590030063008 13796178

[83] Halberstadt AL. Recent advances in the neuropsychopharmacology of serotonergic hallucinogens. Behav Brain Res. (2015) 277:99–120. doi: 10.1016/j.bbr.2014.07.016 25036425 PMC4642895

[84] Isbell H Wolbach A Wikler A Miner E. Cross tolerance between LSD and psilocybin. Psychopharmacologia. (1961) 2:147–59. doi: 10.1007/BF00407974 13717955

[85] Smythies J Sykes E Lord C. Structure-activity relationship studies on mescaline: II. Tolerance and cross-tolerance between mescaline and its analogues in the rat. Psychopharmacologia. (1966) 9:434–46. doi: 10.1007/BF00406453 5998418

[86] Wallach MB Hine B Gershon S. Cross tolerance or tachyphylaxis among various psychotomimetic agents on cats. Eur J Pharmacol. (1974) 29:89–92. doi: 10.1016/0014-2999(74)90174-5 4435048

[87] Winter J. Tolerance to a behavioral effect of lysergic acid diethylamide and cross-tolerance to mescaline in the rat: absence of a metabolic component. J Pharmacol Exp Ther. (1971) 178:625–30. 5571911

[88] Wolbach A Isbell H Miner E. Cross tolerance between mescaline and LSD-25 with a comparison of the mescaline and LSD reactions. Psychopharmacologia. (1962) 3:1–14. doi: 10.1007/BF00413101 14007904

[89] Strassman RJ. Hallucinogenic drugs in psychiatric research and treatment. Perspectives and prospects. J Nerv Ment Dis. (1995) 183:127–38. doi: 10.1097/00005053-199503000-00002 7891058

[90] Brito-da-Costa AM Dias-da-Silva D Gomes NGM Dinis-Oliveira RJ Madureira-Carvalho Á. Toxicokinetics and toxicodynamics of ayahuasca alkaloids N,N-dimethyltryptamine (DMT), harmine, harmaline and tetrahydroharmine: clinical and forensic impact. Pharm (Basel). (2020) 13(1):1–36. doi: 10.3390/ph13110334 PMC7690791 33114119

[91] Cole J Pieper W. The effects of N, N-dimethyltryptamine on operant behavior in squirrel monkeys. Psychopharmacologia. (1973) 29:107–12. doi: 10.1007/BF00422642 4196867

[92] Gillin JC Cannon E Magyar R Schwartz M Wyatt R. Failure of N, N-dimethyltryptamine to evoke tolerance in cats. Biol Psychiatry. (1973) 7:213–20. 4519415

[93] Gillin JC Kaplan J Stillman R Wyatt RJ. The psychedelic model of schizophrenia: the case of N,N-dimethyltryptamine. Am J Psychiatry. (1976) 133:203–8. 10.1176/ajp.133.2.203 1062171

[94] Rosenberg D Isbell H Miner E Logan C. The effect of N, N-dimethyltryptamine in human subjects tolerant to lysergic acid diethylamide. Psychopharmacologia. (1964) 5:217–27. doi: 10.1007/BF00413244 14138757

[95] Strassman RJ. Human psychopharmacology of N,N-dimethyltryptamine. Behav Brain Res. (1996) 73:121–4. doi: 10.1016/0166-4328(96)00081-2 8788488

[96] Halman A Kong G Sarris J Perkins D. Drug–drug interactions involving classic psychedelics: A systematic review. J Psychopharmacol. (2024) 38:3–18. doi: 10.1177/02698811231211219 37982394 PMC10851641

[97] Fontanilla D Johannessen M Hajipour AR Cozzi NV Jackson MB Ruoho AE. The hallucinogen N,N-dimethyltryptamine (DMT) is an endogenous sigma-1 receptor regulator. Science. (2009) 323:934–7. doi: 10.1126/science.1166127 PMC2947205 19213917

[98] Vargas MV Dunlap LE Dong C Carter SJ Tombari RJ Jami SA. Psychedelics promote neuroplasticity through the activation of intracellular 5-HT2A receptors. Science. (2023) 379:700–6. doi: 10.1126/science.adf0435 PMC10108900 36795823

[99] Cornea-Hébert V Riad M Wu C Singh SK Descarries L. Cellular and subcellular distribution of the serotonin 5-HT2A receptor in the central nervous system of adult rat. J Comp Neurol. (1999) 409:187–209. doi: 10.1002/(SICI)1096-9861(19990628)409:2<187::AID-CNE2>3.0.CO;2-P 10379914

[100] Buchborn T Schröder H Dieterich DC Grecksch G Höllt V. Tolerance to LSD and DOB induced shaking behaviour: differential adaptations of frontocortical 5-HT2A and glutamate receptor binding sites. Behav Brain Res. (2015) 281:62–8. doi: 10.1016/j.bbr.2014.12.014 25513973

[101] Gresch PJ Smith RL Barrett RJ Sanders-Bush E. Behavioral tolerance to lysergic acid diethylamide is associated with reduced serotonin-2A receptor signaling in rat cortex. Neuropsychopharmacology. (2005) 30:1693–702. doi: 10.1038/sj.npp.1300711 15756304

[102] Smith RL Barrett RJ Sanders-Bush E. Mechanism of tolerance development to 2, 5-dimethoxy-4-iodoamphetamine in rats: down-regulation of the 5-HT2A, but not 5-HT2C, receptor. Psychopharmacology. (1999) 144:248–54. doi: 10.1007/s002130051000 10435391

[103] Olson DE. Psychoplastogens: A promising class of plasticity-promoting neurotherapeutics. J Exp Neurosci. (2018) 12:1179069518800508. doi: 10.1177/1179069518800508 30262987 PMC6149016

[104] Luan LX Eckernas E Ashton M Rosas FE Uthaug MV Bartha A. Psychological and physiological effects of extended DMT. J Psychopharmacol. (2024) 38:56–67. doi: 10.1177/02698811231196877 37897244 PMC10851633

[105] Tusconi M Fries GR. Neuroprogression in bipolar disorder. Biomarkers Bipolar Disorders: Elsevier. (2022) p:167–89. doi: 10.1016/B978-0-12-821398-8.00009-6

[106] Zhao NO Topolski N Tusconi M Salarda EM Busby CW Lima CN. Blood-brain barrier dysfunction in bipolar disorder: molecular mechanisms and clinical implications. Brain Behav Immunity-Health. (2022) 21:100441. doi: 10.1016/j.bbih.2022.100441 PMC8924633 35308081

[107] Tu SS Kesselheim AS Chao B. Extent of drug patents with terminal disclaimers and obviousness-type double patenting rejections. JAMA. (2024) 332:837–8. doi: 10.1001/jama.2024.14350 PMC11320327 39133468

[108] Holze F Ley L Müller F Becker AM Straumann I Vizeli P. Direct comparison of the acute effects of lysergic acid diethylamide and psilocybin in a double-blind placebo-controlled study in healthy subjects. Neuropsychopharmacology. (2022) 47:1180–7. doi: 10.1038/s41386-022-01297-2 PMC9018810 35217796

[109] Holze F Vizeli P Müller F Ley L Duerig R Varghese N. Distinct acute effects of LSD, MDMA, and d-amphetamine in healthy subjects. Neuropsychopharmacology. (2020) 45:462–71. doi: 10.1038/s41386-019-0569-3 PMC6969135 31733631

[110] Vogt SB Ley L Erne L Straumann I Becker AM Klaiber A. Acute effects of intravenous DMT in a randomized placebo-controlled study in healthy participants. Transl Psychiatry. (2023) 13:172. doi: 10.1038/s41398-023-02477-4 37221177 PMC10206108

[111] Aicher HD Mueller MJ Dornbierer DA Suay D Elsner C Wicki I. Potential therapeutic effects of an ayahuasca-inspired N,N-DMT and harmine formulation: a controlled trial in healthy subjects. Front Psychiatry. (2024) 14. doi: 10.3389/fpsyt.2023.1302559 PMC10804806 38264636

